# Increased *Cdkn2a*, a regulator of cellular senescence, in the PS19 mouse model of tauopathy

**DOI:** 10.1002/alz70855_107206

**Published:** 2025-12-25

**Authors:** Dylan Finneran, Briana G Jackman, Taylor Desjarlais, Dave Morgan, Marcia N. Gordon

**Affiliations:** ^1^ Michigan State University, Grand Rapids, MI, USA

## Abstract

**Background:**

Aging is the primary risk factor for Alzheimer's disease. With aging, senescent cells accumulate within the body, including the brain. Prior studies have identified a broad association between increased levels of cellular senescence and Alzheimer's disease‐like pathology. However, the descriptive literature cannot resolve whether senescent cells result from the pathology or contribute to it. Here, we over‐express the gene products of *Cdkn2a*, a regulator of cellular senescence, in astrocytes of both nontransgenic mice and the PS19 mouse model of tauopathy.

**Method:**

We developed astrocyte‐specific adeno‐associated virus (AAV) serotype 9 vectors expressing both transcripts of the *Cdkn2a* gene under control of the *GFAP* promoter. We injected these vectors bilaterally in the anterior cortex and hippocampus of four‐month‐old nontransgenic (NonTg) or PS19 tauopathy mice with AAV‐GFP serving as a control treatment. Mice survived four months before being behaviorally assessed and euthanized.

**Result:**

We observed a significant effect of tauopathy on several affective behavioral tasks in mice (open field, elevated plus maze, marble burying, and nest building), but this was not altered by over‐expression of *Cdkn2a*. Surprisingly, we did observe a significant improvement in pre‐pulse inhibition of the acoustic startle response in PS19 mice over‐expressing *Cdkn2a* in astrocytes. Finally, we did not observe significant effects of *Cdkn2a* over‐expression on hippocampal dependent learning and memory as measured by the radial arm water maze. At tissue collection, we did not observe significant effects of *Cdkn2a* over‐expression on brain mass but there was a significant main effect of genotype on body mass with PS19 mice having lower body mass than NonTg mice. We are presently measuring markers of senescence (dsDNA breaks and p21 expression), gliosis, tauopathy, and neurodegeneration.

**Conclusion:**

These data indicate selective astrocytic over‐expression of both *Cdkn2a* transcripts has minimal effects on the behavior of NonTg mice. Over‐expression of both *Cdkn2a* transcripts in PS19 mice improved pre‐pulse inhibition but had no impact on other affective behavioral tasks or learning behavioral tasks.

